# Identification of key sentences in a text

**DOI:** 10.3389/frai.2026.1794334

**Published:** 2026-06-23

**Authors:** N. Veer Viswajit, L. Jeganathan, M. Janaki Meena, Jayaram Balabaskaran, Ummity Srinivasa Rao

**Affiliations:** School of Computer Science and Engineering, Vellore Institute of Technology, Chennai, India

**Keywords:** automated evaluation systems (AES), BERT, dynamic thresholding, grid search, key sentence extraction, natural language processing (NLP), SBERT

## Abstract

Human evaluation of students' summative assessments requires significant time and cognitive effort. Existing automated evaluation models struggle to provide accurate evaluations for various reasons. The complexity increases with lengthy student responses, which may contain both relevant and irrelevant information. Therefore, there is a need for an intermediate mechanism to assist human evaluation. To address this gap, this study presents, as a proof of concept, a semantic-aware model called the Key Sentence Identifier (KSI), which extracts sentences that are relevant to the topic and coherent with the meaning conveyed in the student's response. Unlike traditional approaches that rely primarily on keyword matching, KSI employs a dual-embedding framework that integrates transformer-based contextual embeddings (BERT) with semantic similarity-based sentence representations (SBERT). Furthermore, a dedicated dataset has been curated for this task to enable effective training and evaluation of the model. Ablation analysis indicates that the SFT BERT + SFT SBERT configuration achieves the best performance, improving the F1 score from 50.32% (BERT + SBERT) to 86.01%. Furthermore, comparisons with LoRA-based fine-tuning and standard baseline methods show that the proposed KSI model consistently outperforms alternative approaches in identifying contextually relevant sentences. The novelty of the proposed KSI lies in its ability to efficiently evaluate descriptive answers by identifying contextually relevant content for human evaluation. In addition to reducing evaluation time, KSI minimizes the cognitive effort required by human evaluators by eliminating the need to manually identify irrelevant content. Thus, the use of KSI has the potential to enhance the quality of evaluation by enabling evaluators to focus on contextually relevant content while reducing cognitive effort. As improvements in evaluation quality can positively influence learning outcomes, KSI can, more broadly, contribute to the realization of United Nations Sustainable Development Goal 4. A distinguishing feature of the KSI model is that it assists human evaluation and supports integration with future automated evaluation systems.

## 1 Introduction

Assessments are the core component of any teaching-learning process. Assessments measure students' learning throughout the process. Broadly, there are two types of assessments, namely, Formative Assessments (FA) and Summative Assessments (SA). FAs monitor and improve learning during the process, whereas SAs are conducted at the end to measure students' learning. FAs are conducted through objective questions, fill-in-the-blank, etc. The primary aim of SAs is to measure learning at different cognitive levels, such as conceptual clarity, application of learned concepts, and analytical ability. So, SA questions expect subjective answers (open-ended, closed-ended, or both), essays, case studies, and detailed explanations of procedures. In a sense, SAs require detailed subjective responses of considerable length.

Manual evaluation of SAs' responses requires significant quality time from teachers and is cognitively demanding. Studies indicate that, on average, 20% of a teacher's time is devoted to assessment-related activities, leaving less time to mentor students. Therefore, automating SA evaluation may not only be beneficial but also necessary nowadays. Existing tools, such as Moodle and Gradescope, support automated evaluation of SA responses. Recent automated evaluation approaches for SA responses include BERT, LLaMA, and Mistral, as well as hybrid models that combine linguistic features with deep learning. These automated models are primarily built upon techniques from machine learning, natural language processing, and computational linguistics. However, these models still struggle with contextual understanding, semantic variation, and the identification of irrelevant content. Due to these limitations, automated evaluation systems often produce inaccurate or inconsistent evaluations. Hence, there is a need for an intermediate mechanism to assist human evaluation by reducing cognitive load and improving the evaluation process.

The intermediate mechanism to assist human evaluation can be designed in many ways. In that sense, to maximize their scores, students provide lengthy responses that often include irrelevant content. For a question on the description of a “stack” in a data structures course, a student may respond like this: “A stack follows the LIFO principle; stacks are used in function calls and expression evaluation; data structures are very important in computer science and are widely studied in many courses.” Here, the last sentence does not meet the question's expectations and is irrelevant for evaluation purposes. In an SA for three hours with ten questions, for a course with sixty students, even if a student includes a single irrelevant sentence per question, there will be a total of 600 sentences. All six hundred sentences must be read, identified as irrelevant, and ignored by the teacher. This increases the time and effort required for evaluation. This issue is further amplified in large-scale courses such as MOOCs, where the volume of student responses and the number of students are reasonably high. In this context, as a complementary approach to identifying irrelevant content, this study proposes an assistive intermediate mechanism that identifies contextually relevant sentences, referred to as key sentences, in students' SA responses. In a technical sense, this study addresses the problem of “key sentence identification in a text.” Key sentences in a student's response reflect the student's level of understanding, which the teacher can focus on during human evaluation. Although this intermediate mechanism for identifying key sentences is primarily intended to assist human evaluation, it can also support future, more advanced, and more accurate automated evaluation systems by reducing their processing time.

Thus, having established the need for an assistive mechanism for “key sentence identification,” a brief survey of the state-of-the-art techniques reveals that there are no mechanisms for identifying key sentences from an evaluation perspective. Existing key sentence identification models rely on identifying sentences that contain specific words related to the topic or question, referred to as keywords (Hasan and Ng, 2014). However, this approach is limited because it overlooks the sentence's deeper context and semantic meaning (Nomoto, 2022). For example, in a question analyzing the rocks of the Himalayas, the sentence “The Himalayas are a mountain” is not a key sentence. Still, existing methods will identify this sentence as a key sentence because it contains “Himalayas.” Thus, there is a need for a method that identifies key sentences based on their semantic relevance rather than just the presence of keywords. Further, many models address related problems such as summarization and sentence extraction (Nenkova and McKeown, 2012). At this stage, it is important to note that key sentence identification should not be confused with text summarization (Lloret and Palomar, 2012). While text summarization, especially in its abstractive form, condenses the content and may alter the original wording, key sentence identification preserves the student's original sentences and extracts only those that are contextually relevant for evaluation.

Thus, despite the availability of existing approaches, there is currently no feasible solution that effectively addresses the requirements of key sentence identification for evaluation purposes. Existing methods do not adequately ensure the identification of contextually relevant sentences while preserving the original student response. By focusing on sentences that convey the core concepts and context of the student's response, regardless of the exact terminology used, there is a need for a system that can better evaluate the true intent. This highlights a clear research gap and establishes the need to develop specialized models tailored to this problem.

To address this research gap, this paper presents a novel semantically aware model, termed the Key Sentence Identifier (KSI), for identifying and extracting sentences that are both topically relevant and semantically aligned with the meaning expressed in a student's response. To achieve this, KSI employs a dual-embedding framework that integrates transformer-based contextual embeddings (BERT) with semantic similarity-based sentence representations (SBERT). Furthermore, a dedicated dataset has been curated specifically for this task to facilitate effective training and evaluation of the proposed model.

Since this investigation is an initial proof-of-concept study, without any loss of generality, our investigation is limited to the “Operating Systems” domain of Computer Science and Engineering (CSE). The dataset size is kept moderate to demonstrate feasibility rather than large-scale deployment. The current model is not designed for direct generalization across domains; however, the underlying approach remains domain-independent. With appropriately curated data, it can be extended to other domains.

The novelty of the KSI model lies in its assistive role, helping human evaluators by providing relevant content from student responses. Thus, KSI helps evaluators focus more on relevant content, resulting in a higher-quality evaluation of student responses. A quality evaluation leads to quality education. In that broader sense, our KSI can help realize the United Nations Sustainable Development Goal 4.

In Section 2, we provide a detailed literature review on key sentence extraction. Section 3 clearly describes the datasets curated for our study. Section 4 outlines the methodology of the KSI model, along with the required validation metrics. Experiments and the obtained results are discussed in Section 5. In Section 6, we conclude the study by generalizing that the KSI model can be applied to other domains for evaluation using relevant datasets.

## 2 Literature review

In this section, we review several methods for content extraction, including statistical and rule-based approaches and Transformer-based architectures. State-of-the-art techniques can be broadly categorized into two approaches: key sentence extraction and keyphrase extraction.

### 2.1 Extraction of key sentences

The “Extract-then-Evaluate” approach presented in Wu et al. (2024) begins by breaking documents into sentences and then applies a Transformer model to obtain sentence-level embeddings. The employed ranking algorithm operates on these embeddings to extract the most relevant sentences. Key sentence extraction in Reimers and Gurevych (2019) shows how Sentence Transformer models become effective through dataset annotation, which enables them to identify crucial sentences without manual annotation. Liu (2019) introduced BERTSum, which fine-tunes BERT for sentence-level classification to identify and rank important sentences for extractive summarization. Iqbal and Rafi (2015) demonstrated that key sentence extraction relies on keywords according to their findings.

### 2.2 Extraction of keyphrases

Unsupervised and supervised key phrase extraction methods have been extensively studied for extracting keyphrases (groups of keywords). For this study, we review Supervised methods. Early keyphrase extraction methods fall under statistical and machine learning approaches, as evidenced by KEA (Witten et al., 1999), which used binary classification with TF-IDF and keyphraseness; Maui (Medelyan et al., 2009), which leveraged semantic context; and GenEx (Turney, 2000), which further employed decision trees. For key sentence extraction, connectivity-based approaches include (Okazaki et al., 2005), which uses spreading activation; Yu (2010), which focuses on connectives; and Wang et al. (2012), which employs a Wikipedia-based graph to link sentences to keywords. Dependency graph models are another subtopic, with Gong and Liu (2001) introducing a generic text summarization method based on a relevance measure and latent semantic analysis. The triangle analysis of dependency graphs, as suggested in Cheng et al. (2013), adds syntactic features. Advancements in key sentence extraction have been made through the incorporation of Transformer models and Large Language Models (LLMs).

Witten et al. (1999) and Medelyan et al. (2009) were the first supervised key sentence extraction methods that employed TF-IDF, along with first occurrence and key phraseness measures using Naive Bayes and decision trees classifiers. The key phrase extraction method CeKE (Caragea et al., 2014) improved the efficiency of its process by incorporating context-dependent features from the citation and external sources. Supervised ranking methods, such as Ranking SVM (Jiang et al., 2009) and MIKE (Zhang et al., 2017), learn to rank candidate phrases, considering linguistic and semantic features. (Meng et al., 2017) used semantic context, but (Chen et al., 2018) further improved performance by incorporating correlation constraints to increase diversity. Gollapalli et al. (2017) achieved unique phrase detection by merging TF-IDF and TextRank.

Some of the Transformer-based approaches for keyphrase extraction include (Bsir and Zrigui, 2024), which fine-tuned XLM-RoBERTa on a large Arabic corpus for keyword extraction. Doostmohammadi et al. (2019) introduced a sequence-to-sequence model for keyphrase extraction from Persian news articles. Huang et al. (2022) demonstrated that LLMs can perform well in keyphrase extraction through model feedback. The Topic-based Adversarial Neural Network (TANN) is used to transfer knowledge between domains. With the preceding and the succeeding word context, Basaldella et al. (2018) developed a Bi-LSTM RNN model. Alzaidy et al. (2019) connected Bi-LSTM with Conditional Random Fields (CRF) for output dependency detection.

### 2.3 LLM-based approaches for extractive summarization

LLMs have made rapid advancements in text generation and summarization. Summarization is achieved by paraphrasing or by combining sentences.

DeepExtract (Onan and Alhumyani, 2024) is a semantic-based sentence extraction approach that incorporates contextual and structural cues from documents. StrucSum employs structure-aware prompting strategies that integrate graph representations into LLM reasoning (Yuan et al., 2025).

LLM-based models perform better in abstractive settings and are less reliable for extractive summarization, particularly for long documents (Zhang et al., 2023). They may introduce hallucinated content. Further, LLM-based models rely on complex prompting strategies.

While LLM-based methods are promising, their limitations highlight the need for alternative extractive approaches.

### 2.4 Large concept models

Traditional Large Language Models (LLMs) process language as sequences of tokens or words and predict the next token during generation. Conversely, the Large Concept Model (LCM) (LCM Team et al., 2024) attempts to perform language modeling in a sentence representation or concept space. Instead of treating words independently, LCM converts entire sentences into semantic embeddings called concept representations and models transitions between these concepts. The functionality of the LCM framework can be understood through the following example paragraph *P* consisting of ten sentences.

#### 2.4.1 Example paragraph P

“Dynamic programming solves optimization problems. Memoization stores intermediate results. Previously computed values are reused efficiently. Arrays are commonly used in implementations. The weather was pleasant yesterday. Fibonacci numbers can be computed efficiently. Bottom-up methods use iteration. Space complexity can sometimes be reduced. Dynamic programming follows optimal substructure. All is well.”

Assume that the sentences in paragraph *P* are denoted sequentially as *S*_1_, *S*_2_, …, *S*_10_.

In the LCM framework, each sentence is transformed into a semantic concept vector. The first sentence *S*_1_ is transformed into a concept vector *C*_1_, the second sentence *S*_2_ into *C*_2_, and so on. Here, *C*_1_, *C*_2_, …, *C*_10_ represent semantic embeddings corresponding to the ten sentences in the paragraph.

The transformation process may be represented as follows:

$$S_{1}\to C_{1},S_{2}\to C_{2},\ldots ,S_{10}\to C_{10}$$

where each *C*_*i*_ denotes the semantic concept representation corresponding to sentence *S*_*i*_.

The LCM framework learns semantic relationships and transitions among these concept representations, thereby capturing the semantic flow of ideas at a conceptual level rather than relying solely on next-token prediction. In the concept representation space learned by LCM, sentences discussing similar ideas tend to appear closer together, whereas unrelated sentences may appear farther apart.

For the above paragraph *P*, the concept representations corresponding to *S*_1_, *S*_2_, and *S*_3_ may appear conceptually related because these sentences discuss the core ideas of dynamic programming, such as optimization, memoization, and reuse of previously computed values. Sentence *S*_6_ may also appear semantically connected since Fibonacci computation is a classical application of dynamic programming. Similarly, *S*_7_, *S*_8_, and *S*_9_ may remain conceptually associated because they discuss iterative computation, space optimization, and optimal substructure properties of dynamic programming. Sentence *S*_4_ may appear moderately related because it refers to implementation aspects using arrays. Conversely, sentence *S*_5_ (“The weather was pleasant yesterday.”) introduces a completely unrelated topic and therefore may appear far away from the dynamic programming concept cluster in the semantic space. Similarly, sentence *S*_10_ (“All are well.”) does not provide any meaningful contextual information related to the technical discussion and may therefore behave as another semantic outlier. Thus, LCM focuses on modeling semantic relationships and conceptual transitions in a continuous representation space.

The proposed KSI model is designed to assist the human evaluation of students' answers in examinations. The objective of KSI is to identify semantically relevant sentences from student responses. Given the above paragraph *P*, KSI filters out *S*_5_ and *S*_10_ as contextually irrelevant sentences and returns *S*_1_, *S*_2_, *S*_3_, *S*_4_, *S*_6_, *S*_7_, *S*_8_, and *S*_9_ for further human evaluation. Thus, KSI operates at the sentence level and performs semantic filtering to assist automated or human evaluation.

Although both LCM and KSI operate at a semantic sentence level, their objectives are fundamentally different. LCM is primarily a large-scale generative framework designed for concept-level language modeling, reasoning, and generation. KSI explicitly identifies relevant sentences while discarding irrelevant content. Thus, KSI can be viewed as an application-oriented semantic filtering framework for educational assessment, whereas LCM is a general-purpose concept-space language modeling architecture.

Although Large Concept Models possess semantic understanding capabilities that may indirectly support key sentence identification, they are not explicitly designed for educational key sentence extraction. To use LCM for key sentence identification, additional task-specific mechanisms, such as relevance scoring or classification layers, would still be required to identify relevant sentences in student responses. Conversely, the proposed KSI model specifically targets the identification of relevant sentences from student responses to assist human evaluation.

## 3 Dataset

Since our study is a proof-of-concept, we chose the Operating Systems domain for dataset curation. Accordingly, the dataset size is kept small. While the current implementation may not be directly generalizable or reproducible in its present form, the underlying methodology is, in principle, domain-independent and can be extended to other domains with appropriate data. We curate two datasets: one for keyword identification and another for labeling sentences as key or not.

### 3.1 Dataset for identifying keywords

We curate a dataset of paragraphs from the Operating Systems domain, each annotated with its keywords. Operating System (OS) concepts were chosen for this study due to their varied qualities, such as their organized structure and the presence of both descriptive and analytical answers. Evaluating student responses in this field presents obstacles, as answers may involve technical depth and multiple interpretations of the same concept. Evaluating open-ended responses on memory management and concurrency demands significant time and manual effort from instructors, as the process remains highly subjective.

Each paragraph was made up of five sentences, carefully chosen to have both conceptually relevant and irrelevant sentences. The relevant and irrelevant sentences can occur in any position of the paragraph. For this purpose, multiple variants of each paragraph were generated by randomly rearranging the sentences. This rearranging is required to train the model on semantics. The dataset was annotated with the relevant keywords. 80% of the dataset is used for training. To ensure academic rigor, we employed ChatGPT 4.0 and DeepSeek R1 to generate and validate the paragraph content for accuracy and reliability. Table 1 depicts the composition of the dataset.

**Table 1:** The composition of the keyword dataset.

Paragraph	Composition
Paragraph 1	5 relevant sentences
Paragraph 2	4 relevant sentences and 1 irrelevant sentence
Paragraph 3	3 relevant sentences and 2 irrelevant sentences (either from the same irrelevant domain or separate irrelevant domains)
Paragraph 4	2 relevant sentences and 3 irrelevant sentences (either from the same irrelevant domain or separate irrelevant domains or a combination of both types)
Paragraph 5	1 relevant sentence and 4 irrelevant sentences (either from the same irrelevant domain or separate irrelevant domains or a combination of both types)

The keyword extraction dataset consists of 1,015 unique paragraphs derived from the OS domain content, each annotated with relevant keywords. Five variants of each paragraph were generated. In total, the dataset has 5,075 paragraphs.

### 3.2 Dataset for sentence labeling

We curate a dataset with binary labels to fine-tune our process for identifying relevant sentences in a given paragraph. Various sentences were collected from the Operating Systems domain across multiple sources. We analyzed each sentence, and the relevant sentences were assigned the label 1 and the irrelevant sentences the label 0. For SBERT fine-tuning, input pairs were created by combining positive pairs and negative pairs. The dataset was organized into two columns: Sentence and Label. Table 2 presents a few sample entries from the dataset.

**Table 2:** Example sentences from dataset.

Sentence	Label
“*File systems organize, store, and retrieve data on storage devices within an OS.”*	1
“*Process scheduling in operating systems ensures efficient execution of multiple programs concurrently.”*	1
“*Computer networks enable global communication using protocols such as TCP/IP and HTTP.”*	0
“*Distributed systems rely on networked components to achieve fault tolerance and scalability.”*	0

For sentence-level classification, we constructed a dataset of 2,700 unique sentences, each labeled as either relevant or irrelevant to the OS domain, with assistance from LLMs. The dataset is balanced to ensure equal representation of both classes, which supports unbiased training and reliable evaluation of the classification model.

### 3.3 Rationale for custom dataset construction

Existing benchmark datasets for extractive summarization, such as CNN/DailyMail and DUC, primarily provide sentence-level summaries without explicit keyword annotations. However, the proposed KSI framework follows a two-phase pipeline: keyword extraction followed by sentence selection, which requires both keyword- and sentence-level supervision. Since such aligned annotations are not available in standard benchmark datasets, directly applying them would not allow for a complete evaluation of the proposed approach. Therefore, a custom dataset was constructed to jointly capture keyword relevance and sentence importance, enabling a comprehensive assessment of the pipeline as a whole.

## 4 Methodology of key sentence identifier

**Figure 1:**
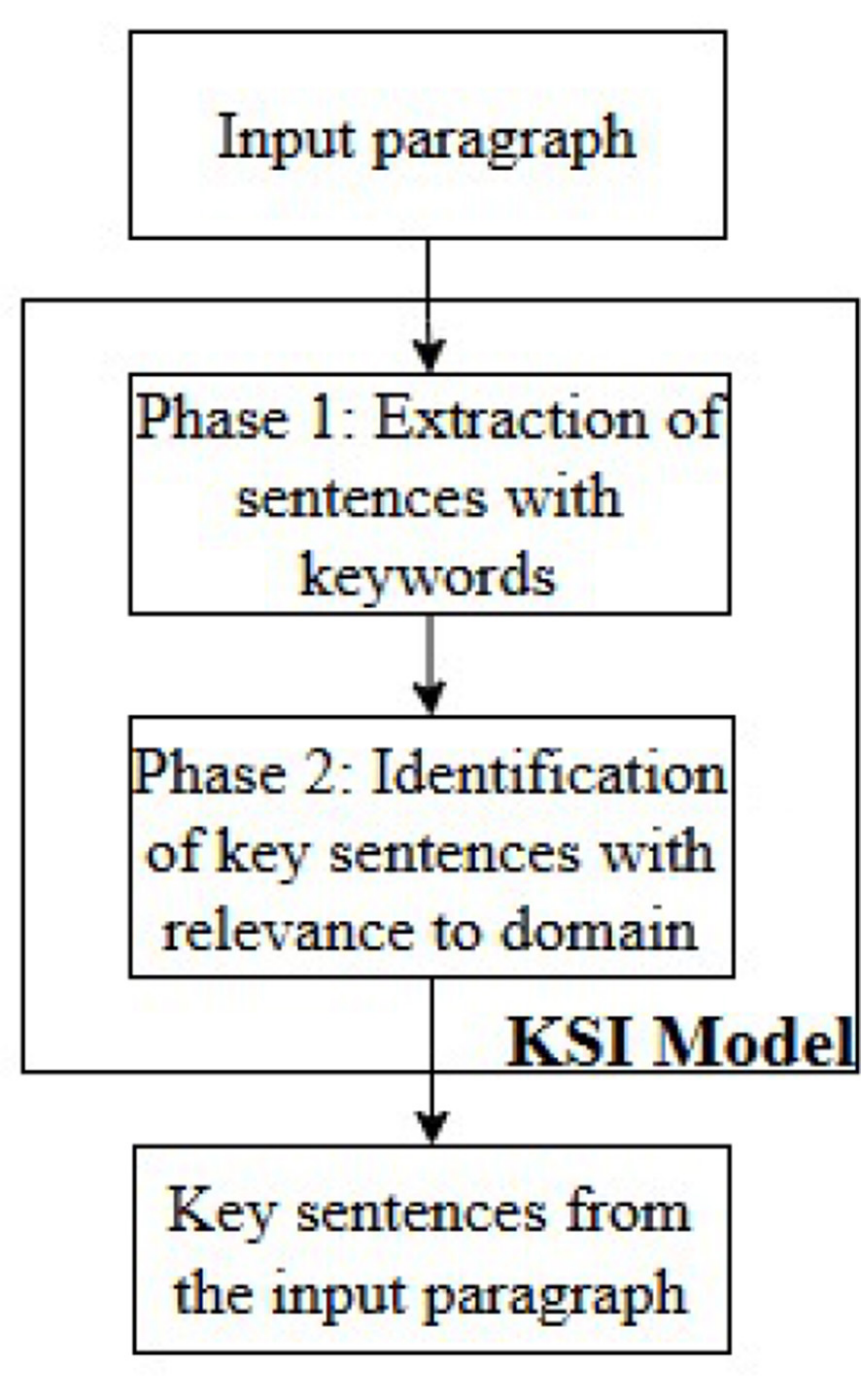
The workflow of the KSI model.

We propose a novel model, called the KSI, based on the premise that key sentence extraction can be achieved effectively through a two-phase process. In the first stage, we use a domain-specific dataset of paragraphs with annotated keywords to fine-tune a transformer-based model for keyword extraction via direct Supervised Fine-Tuning (SFT). In the second stage, a separate transformer model is fine-tuned for sentence-level classification on a dataset in which sentences are labeled for their relevance to the domain. In this stage, we first embed the candidate sentences obtained in stage 1 using BERT and SBERT. We next compute an average for each candidate sentence's embeddings so that the resulting averages reflect contextual and semantic relevance to the domain. Refer to these averages as average embeddings of candidate sentences. We then consider a small sample of reference sentences (relevant to the domain) from the curated dataset and perform the same task to compute their average embeddings. We then compute a cumulative average of these reference sentences, which we refer to as the cumulative average embedding. We then compute the cosine similarity between each of the average embeddings of candidate sentences and the cumulative average embedding of reference sentences. Based on the similarity score, key sentences are identified using an appropriate dynamic threshold. The dynamic threshold value derived from a grid search helps separate key sentences from non-key sentences. Thus, our overall pipeline contains two main phases: Sentence Extraction with Keywords, and sentence-level classification that leads to key sentence identification. This pipeline is described in the Figure 1.

### 4.1 Phase-1: sentence extraction with keywords

**Figure 2:**
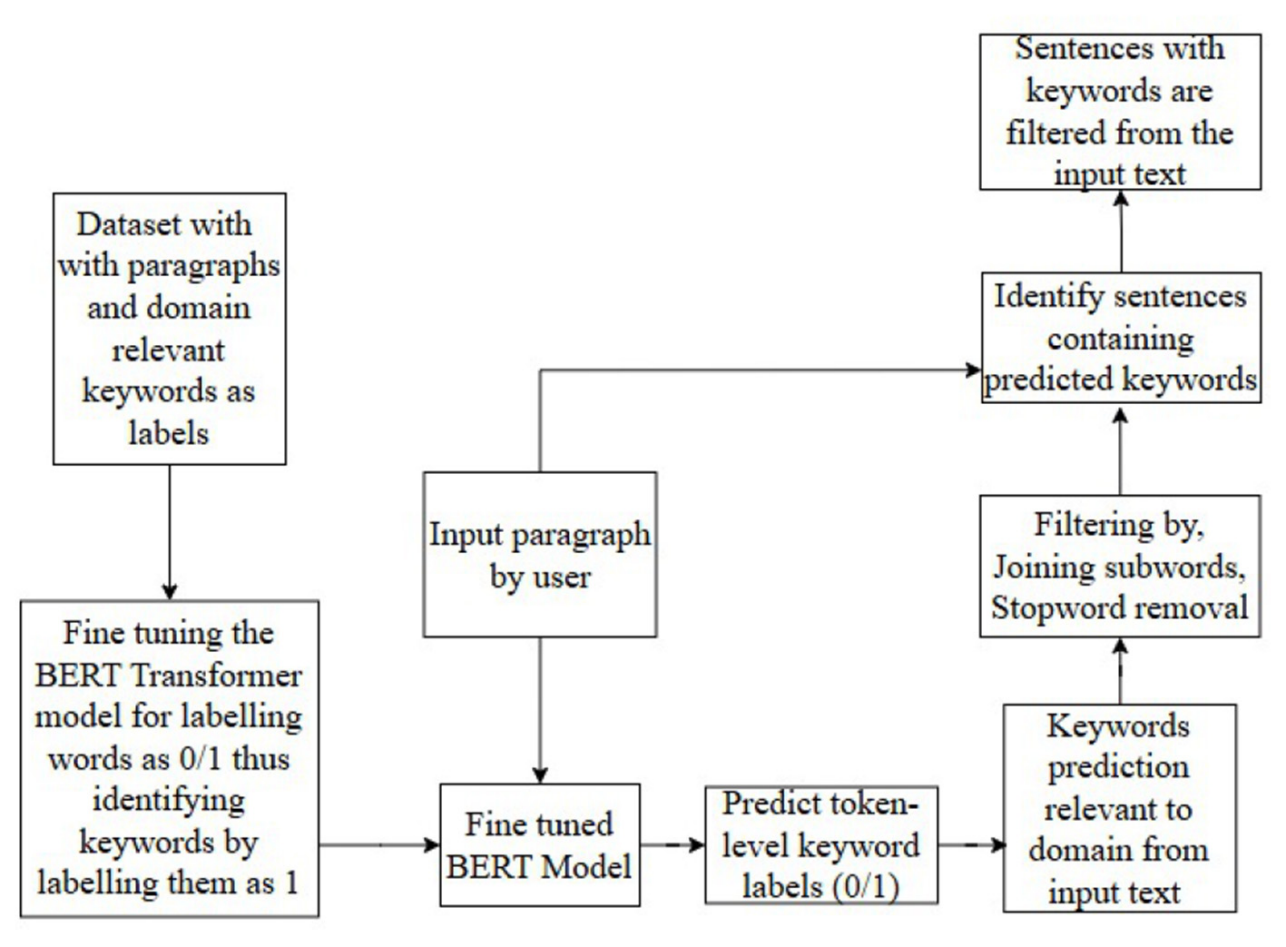
Sentence extraction with keywords.

This section presents a method to identify keywords specific to a particular domain in the input text and, consequently, extract the sentences in which those keywords appear. The entire workflow for this stage is depicted in Figure 2.

#### 4.1.1 Choice of transformer model for keyword identification

The research analyzes various transformer-based architectures for extracting keywords from text. We assess different model types via supervised fine-tuning and evaluate them using accuracy metrics. The comparative assessment based on accuracy guides the selection of the extraction model that balances performance and computational efficiency.

The BERT model (Devlin et al., 2019) was selected for this study after a comparative evaluation with RoBERTa and SBERT. During fine-tuning, BERT outperformed the others in accurately identifying keywords from sentences in the OS domain. The keyword extraction task is formulated as a token-level classification task, where each token in a sentence is assigned a binary label indicating whether it is a keyword (1) or not (0). This is mainly because BERT uses Masked-Language-Modeling (MLM) and Next-Sentence-Prediction (NSP) to capture contextual dependencies between tokens, which makes it perform extremely well on token classification tasks and thus a good choice for keyword extraction. RoBERTa better represents contextual information because it is trained on larger datasets, but it misses token dependencies since it uses only MLM, not NSP. Furthermore, RoBERTa lacks the token classification optimizations found in BERT. SBERT is better suited to sentence-level tasks than to identifying individual keywords, as it generates sentence embeddings that capture semantics well; it is not optimized for token-level classification. Table 3 presents a comparative analysis of BERT, RoBERTa, and SBERT, which prompted us to choose BERT for our study.

**Table 3:** Comparison of the model's performance on keyword prediction.

Pre-trained model	Accuracy after 3^*rd*^ epoch
BERT (bert-base-uncased)	96.72%
RoBERTa (roberta-base)	94.23%
SBERT (sentence transformers/paraphrase- multilingual-MiniLM-L12-v2)	91.34%

After dataset creation, text data is preprocessed to standardize it and ensure effective model training and precise keyword prediction. During training, the BERT tokenizer transforms paragraphs into standardized sequences of equal length through padding or truncation. The model ignores both padding tokens and non-keyword terms as they occur, focusing on labeling relevant keywords. The dataset is split into training and testing sets. The text processing, which includes lemmatization, punctuation removal, and stopword removal, produces uniform sequences that help the model fine-tune its keyword identification while minimizing noise.

Then, the pre-trained BERT model is fine-tuned to identify keywords relevant to the Operating Systems (OS) domain from textual data. The fine-tuning process uses a labeled dataset containing keywords associated with various sentences.

The fine-tuned model then takes in a user-provided input paragraph and predicts token-level keyword labels. The prediction process is followed by merging sub-word tokens, and filtering steps (lemmatization and stop-word removal) are performed to produce the final list of domain-relevant keywords. These keywords are then traced back to the original paragraph to identify the sentences that contain them. Finally, the sentences containing one or more of the predicted keywords are extracted.

### 4.2 Phase-2: identification of key sentences with relevance to domain

**Figure 3:**
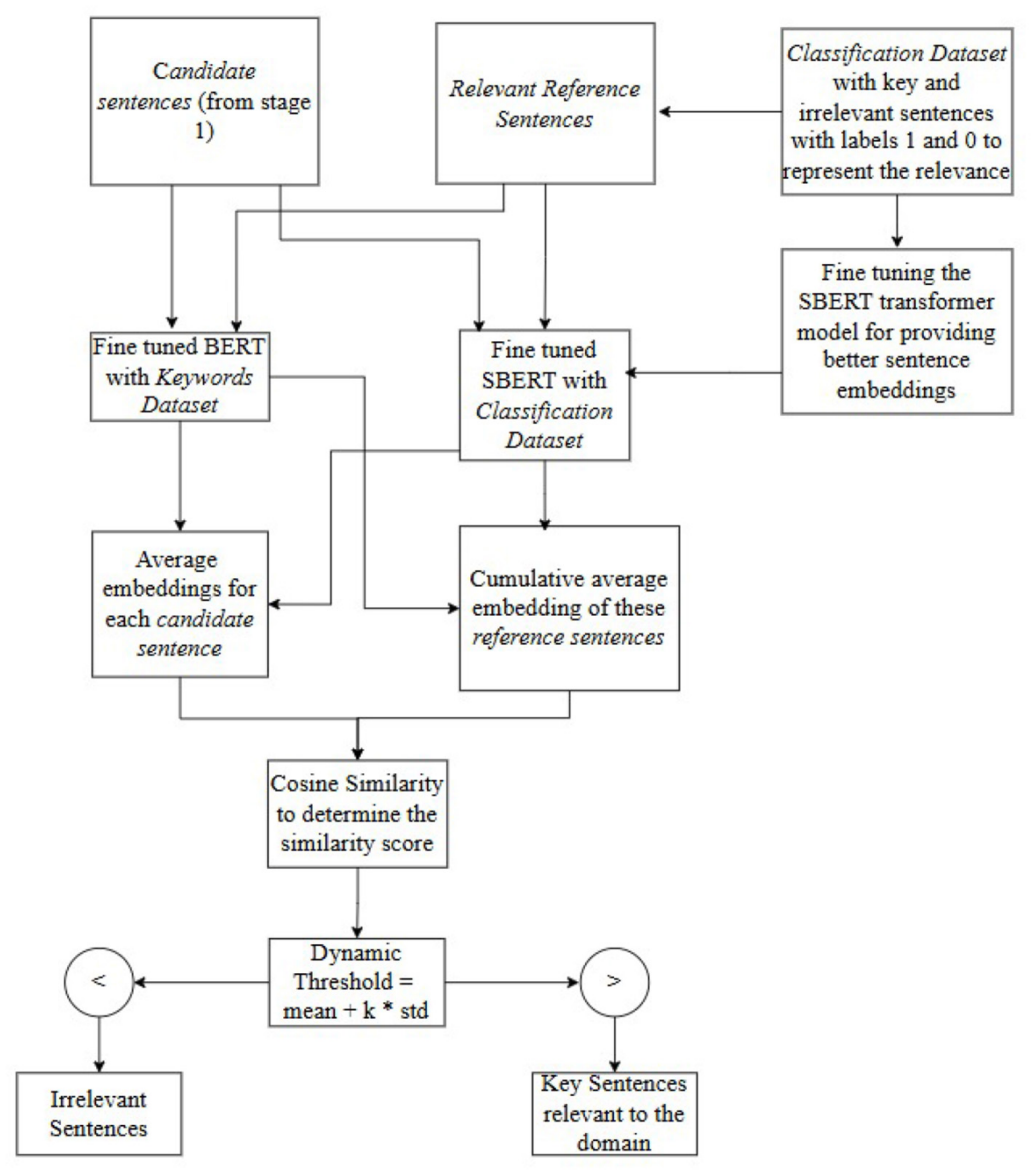
Identification of key sentences relevant to domain.

In the first phase of the KSI model, we have extracted the sentences that have context-aware keywords from the given input paragraph. Next, in the second phase of the KSI model, we identify sentences with keywords that reflect contextual and semantic relevance to the domain. We achieve this through a combination of BERT and the Sentence Transformer model that obtains token-level contextual embeddings from BERT and richer sentence-level semantic representations from the Sentence Transformer. Figure 3 depicts the overall workflow of the second stage of the KSI model.

#### 4.2.1 Choosing the transformer model for sentence classification

After curating the sentence-labeling dataset, the SBERT model was fine-tuned for sentence-level classification and similarity tasks in the Operating Systems (OS) domain to generate robust sentence embeddings tailored to our dataset.

We evaluated several SBERT variants, including MPNet-based (all-mpnet-base-v2) and DistilRoBERTa-based (all-distilroberta-base-v1) models, using Online Contrastive Loss with tuned hyperparameters to ensure stable optimization and prevent overfitting. Even though validation experiments did not significantly differentiate the performance, the benchmark results of sbert.net revealed significant trade-offs. Although all-mpnet-base-v2 has the best STS (69.57), it is much slower (2,800 sentences/sec) and heavier (420 MB). All-distill-roberta-base-v1 has medium STS performance (68.73) but with increased latency and memory costs (4,000 sentences/sec, 290 MB). On the contrary, all-MiniLM-L6-v2 offers a high STS score (68.06) and is much more efficient (14,200 sentences/sec, 80 MB). Considering semantic performance, computational overhead, memory footprint, and inference speed, all-MiniLM-L6-v2 delivered the best balance and consistently performed well during fine-tuning for OS-domain sentence-level semantic relevance classification (Reimers and Gurevych, 2019).

The fine-tuning process employed direct fine-tuning (SFT) with contrastive learning, using input sentence pairs generated from OS textbook-derived content. Positive pairs paired two OS-relevant sentences, while negative pairs paired one OS-relevant sentence with one irrelevant OS-sentence.

We use both fine-tuned BERT (from Phase 1) and SBERT models to generate embeddings for sentences containing the extracted keywords. We then average these embeddings to create a final representation for each sentence. Averaging embeddings generated by the BERT and SBERT models, after ensuring dimensional alignment, yields a combined contextual analysis that retains semantic understanding, enabling better assessment of the significance of important sentences.

#### 4.2.2 Embedding fusion strategy

In the proposed second phase of our KSI model, we combine the representations from BERT and SBERT (Reimers and Gurevych, 2019) to identify sentences that capture both contextuality and semanticity. For this purpose, we propose a unified sentence representation by combining the two embeddings.

An exploration of existing embedding fusion strategies was carried out. Linear fusion methods, such as averaging, have been widely used in embedding ensembles due to their simplicity, computational efficiency, and ability to reduce model-specific noise (Muromägi et al., 2017). Other fusion strategies, such as attention-based approaches, were also explored. Since our investigation is of a Proof-of-Concept nature, average fusion was chosen.

We adopt a simple average-based fusion strategy:

$$E_{\text{combined}}=\frac{E_{\text{BERT}}+E_{\text{SBERT}}}{2}$$

This average fusion strategy captures the strengths of both contextual and semantic representations.

#### 4.2.3 Key sentence extraction

The section focuses on identifying important sentences in a particular domain that contain the most significant information.

Phase 1 (Figure 2) of the extraction process starts with identifying sentences that include relevant keywords to the domain.

After choosing an appropriate model, we first embed the candidate sentences obtained in phase 1 using BERT and SBERT. We next compute averages for each candidate sentence's embeddings, so that the resulting averages reflect contextual and semantic relevance to the domain, which we refer to as the average embeddings of candidate sentences. We then consider a small sample of reference sentences (relevant to the domain) from the curated dataset and perform the same task to compute average embeddings for them. We then compute a cumulative average of these reference sentences, which we refer to as the cumulative average embedding. We then compute the cosine similarity between each of the average embeddings of candidate sentences and the cumulative average embedding of reference sentences. Based on the similarity score, key sentences are identified using an appropriate dynamic threshold. The dynamic threshold value derived from a grid search helps separate key sentences from non-key sentences. Thus, Phase 2 (Figure 3) uses a hybrid embedding system that compares contextual sentence relevance by averaging fine-tuned BERT and SBERT embeddings.

### 4.3 Dynamic thresholding technique

Fixed threshold approaches are often inadequate for key sentence identification, as similarity score distributions vary across paragraphs with different sentence compositions. In the proposed pipeline, Phase 1 generates candidate sentences, which are then embedded and processed in Phase 2 to compute similarity scores relative to reference sentence representations. A thresholding mechanism is therefore required to distinguish relevant sentences from irrelevant ones. In that sense, we propose a dynamic thresholding mechanism that adapts to the distribution of similarity scores within each paragraph.

For each sentence, we compute how similar it is to a reference representation. Then, for all sentences in a paragraph, we analyze the distribution of these similarity scores using their average (μ) and standard deviation (σ). Based on the analysis, we decide which sentences are relevant (Li and Liu, 2009).

The relevant threshold is defined as: *T* = μ+*kσ*. A sentence in a paragraph is classified as a relevant key sentence if its similarity score is greater than the dynamically computed threshold *T* for that paragraph. Here, the parameter *k* controls the threshold.

To determine the optimal value of *k*, a grid search is performed over the range [−2, 2] with increments of 0.05. For each candidate value of *k*, a score, *Score*(*k*), the difference between the average similarity score of relevant sentences and the average similarity score of irrelevant sentences in that paragraph, is computed.

$$\text{Score}\left(k\right)=\frac{1}{\left|R\right|}\underset{i\in R}{\sum }s_{i}-\frac{1}{\left|I\right|}\underset{j\in I}{\sum }s_{j}$$

where *R* and *I* denote the sets of sentences classified as relevant and irrelevant, respectively. We choose the *k* that has the maximum *Score*(*k*) for that paragraph. Based on the chosen *k*, the threshold *T* is calculated for the paragraph. The threshold is computed for each paragraph, allowing the model to adapt to that paragraph's similarity distribution. The choice of *k* maximizes the separation of relevant key sentences and the irrelevant sentences in that paragraph.

### 4.4 Evaluation metrics

We evaluate our model using standard classification metrics: Accuracy, Precision, Recall, and the F1 score. These metrics assess the semantic relevance of the key sentences generated by our KSI model. Accuracy provides an overall measure of correct classification. Precision reflects the model's ability to avoid wrong classification. Recall measures the proportion of true key sentences that are correctly classified. The F1 score (Sokolova and Lapalme, 2009) balances precision and recall. Furthermore, the cosine similarity metric is used to measure the semantic similarity between the generated and reference sentences. Cosine similarity is commonly used in BERT-based models (Reimers and Gurevych, 2019).

## 5 Experimentation and results

In this section, we describe training the KSI model on the curated datasets.

### 5.1 Experimentation on phase-1 of KSI model

For the keyword extraction task, we used the BERT model (bert-base-uncased), fine-tuned via token classification, from the Hugging Face Transformers library.

#### 5.1.1 BERT fine-tuning and model evaluation in phase-1

**Figure 4:**
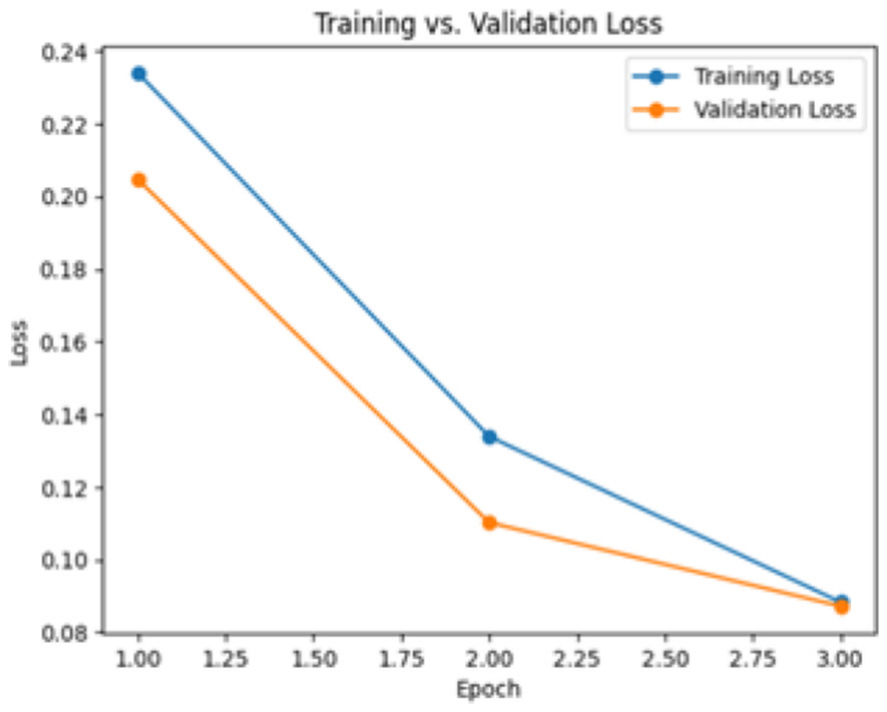
Training vs. validation loss curve for SFT BERT.

The hyperparameters used for fine-tuning BERT in the Google Colab environment are given in Table 4. Training was performed with the AdamW optimizer, and performance was monitored by analyzing loss curves (Figure 4) and evaluating precision, recall, accuracy, and F1 score. The model demonstrates effective learning ability because its training vs. validation loss curves show decreasing values that converge, with no evidence of overfitting or underfitting. By the third epoch, as in Table 5, both losses are closely aligned, suggesting good generalization. The model demonstrates substantial improvements in precision, recall, and F1 scores across its epochs, achieving an exceptional F1 score above 0.91, as shown in Table 5.

**Table 4:** Hyperparameters of fine-tuning BERT.

Hyperparameters	Value
Learning rate	1e-5
Hidden dropout	30%
Weight decay	0.005
Warmup steps	500
Epochs	3
Batch size	8

**Table 5:** Training metrics over 3 epochs for fine-tuning BERT.

Epoch	Training loss	Validation loss	Accuracy	Precision	Recall	F1 score
1	0.234000	0.204686	0.907815	0.752894	0.768913	0.760819
2	0.133900	0.110209	0.953964	0.846798	0.926123	0.884686
3	0.088300	0.087191	0.965572	0.863815	0.972813	0.915080

The model shows an increase in metric values, thereby improving OS-relevant keyword identification while minimizing both false positives and false negatives.

Overall, these results show that the fine-tuned BERT model has reliable capabilities in extracting keywords in Phase 1 of the KSI Model.

### 5.2 Dynamic thresholding mechanism

The parameter k in the dynamic thresholding mechanism is tuned via a grid search over the range [–2, 2] with increments of 0.05. The search range was empirically chosen based on preliminary experiments, which showed that values outside this interval did not improve class separability. For each value of k, the corresponding threshold is computed and used to classify sentences as relevant or irrelevant. The optimal value is selected based on its ability to maximize the separation between relevant and irrelevant sentences.

### 5.3 Experimentation on phase-2 of the KSI model

In this phase, we classify the key sentences obtained in Phase 1 based on both contextual and semantic relevance. Here for sentence-level classification, we fine-tuned SBERT using a contrastive learning approach with chosen hyperparameters as given in Table 6.

**Table 6:** Hyperparameters of fine-tuning SBERT.

Hyperparameters	Value
Learning rate	1e-5
Hidden dropout	30%
Weight decay	0.005
Warmup proportions	0.1
Epochs	5
Batch size	16

#### 5.3.1 SBERT model selection and fine-tuning performance

**Figure 5:**
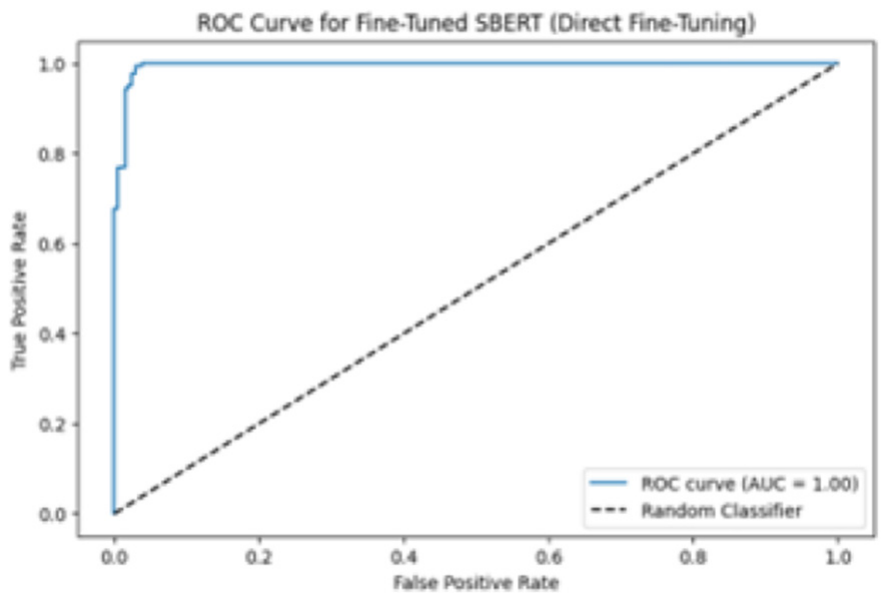
ROC curve for SBERT SFT.

**Figure 6:**
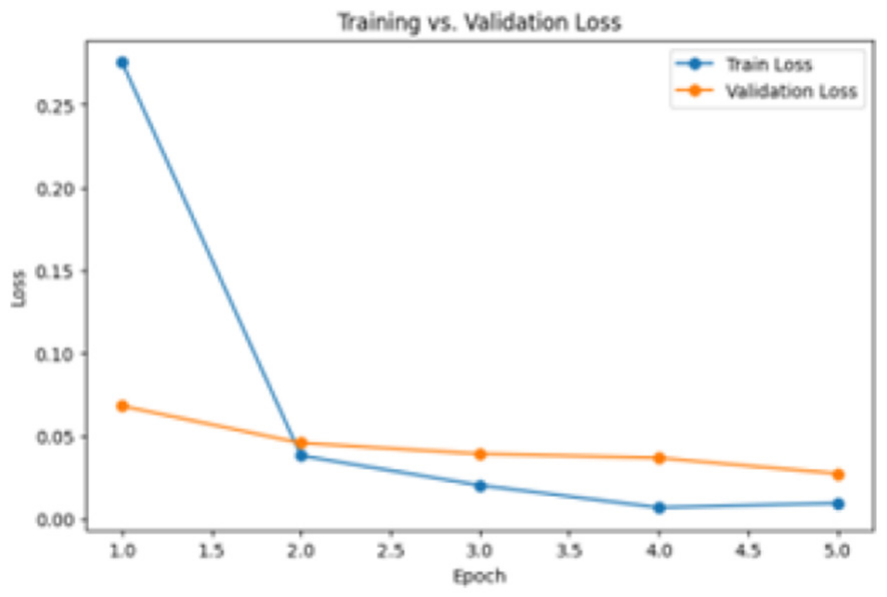
Training vs. validation loss for SBERT SFT.

The direct fine-tuning (SFT) approach for SBERT exhibits near-perfect classification capabilities, as evidenced by the AUROC value being 99.47 (Figure 5). The training and validation loss curves (Figure 6) converge to very low values by the final epoch, suggesting that the model has effectively learned to discriminate between OS-relevant and irrelevant sentences with minimal overfitting.

This strong alignment between losses and the high AUROC underscores the robustness and precision of the fine-tuned SBERT for key sentence identification, as shown in Figures 5, 6. The findings demonstrate encouraging performance measures from the SBERT model adaptation while avoiding overfitting since the current training data seems to be a relatively straightforward classification context. The model requires more extensive and diverse data to achieve better performance and advancement.

### 5.4 Results and analysis of KSI model

We achieve optimal results by exploring different k values until we find the threshold that accurately represents the natural score distinctions between paragraphs. The system labels sentences that exceed this adaptive threshold as key. The final paragraph classification is represented through a binary string, such as say "11001", to indicate significant sentence positions.

**Figure 7:**
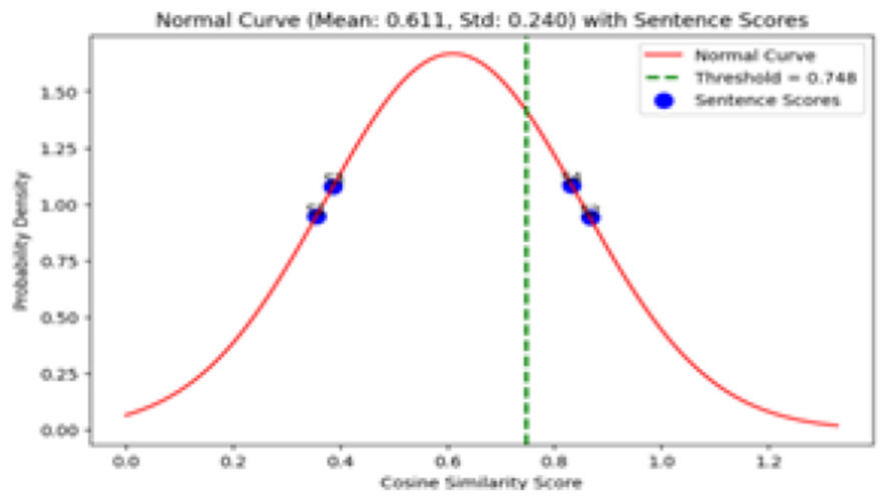
Example of a paragraph's sentence distribution.

**Example:** “*The accurate transmission of data packets between computer devices depends on routers as fundamental network components which direct information between devices. Operating systems provide a graphical user interface (GUI) that enhances user interaction with the computer, while the kernel handles core tasks such as process management and device control. Network protocols like TCP/IP establish the rules for data transmission, facilitating reliable communication across the internet. Operating systems manage system memory, enabling multiple programs to run smoothly without interference. In space missions, scheduling helps manage astronaut activities on a spacecraft.”*. **Output:** Best k: 0.55, Dynamic Threshold: 0.7480412125587463, Predicted Label: 01010. The diagrammatical representation of the example is shown in Figure 7. Table 7 shows the similarity scores of the sentences.

**Table 7:** Sentences with keywords and their scores.

Sentences	Scores after cosine similarity
“The accurate transmission of data packets between devices depends on routers as fundamental network components which direct information between devices.”	0.3558751
“Operating systems provide a graphical user interface (GUI) that enhances user interactions with the computer, and the kernel handles core tasks like process management and device control.”	0.8665799
“Network protocols like TCP/IP establish the rules for data transmission, facilitating reliable communication across the internet.”	0.3873144
“Operating systems manage system memory, enabling multiple programs to run smoothly without interference.”	0.8323955

#### 5.4.1 Validation of the two-phase pipeline of KSI model

In the validation phase, we compared several pipeline combinations to assess the overall effectiveness of key sentence identification. The comparative results of the various variants used in the pipelines have shown some distinct performance variation. The lowest scores across all metrics are for the baseline BERT + SBERT configuration, which demonstrates the drawbacks of identifying domain-specific key sentences using unfine-tuned models. The addition of supervised fine-tuning to either BERT (SFT BERT + SBERT) or SBERT (BERT + SFT SBERT) results in significant improvements in accuracy, precision, recall, and F1 score, and suggests the relevance of training each model on OS-specific data. Of all the considered variants, the most fine-tuned one, SFT BERT + SFT SBERT (KSI), achieves the highest overall performance and the best values across all evaluation metrics. The summary of these results in Table 8 supports the idea that the two-phase fine-tuning pipeline is the most effective system of domain-specific key sentence identification.

**Table 8:** Comparison of different pipelines.

Evaluation metrics	BERT + SBERT	SFT BERT + SBERT	BERT + SFT SBERT	SFT BERT + SFT SBERT (KSI)
Accuracy	58.29%	79.79%	78.57%	84.29%
Precision	0.7696	0.9227	0.9065	0.9501
Recall	0.3738	0.7234	0.7465	0.7853
F1 score	0.5032	0.8115	0.8187	0.8601
Mean cosine similarity	0.4143	0.7737	0.7986	0.8386

#### 5.4.2 LoRA-based fine-tuning and performance analysis

As suggested in Hu et al. (2022), low-rank modifications to LoRA within a pre-trained model reduce the number of trainable parameters, making the training process more efficient with minimal memory. At the same time, these modifications preserve the model's performance. In our KSI model, we fine-tuned the SBERT to identify context-aware sentences among the candidate sentences. Based on this suggestion, we compare the performance of the standard fine-tuned SBERT in the KSI model with that of the LoRA-fine-tuned SBERT.

LoRA was applied to the SBERT model (paraphrase-MiniLM-L6-v2) with a rank of *r* = 8, scaling factor α = 32, and dropout of 0.1, targeting the attention projection layers (query, key, value). This configuration provides a balance between parameter efficiency and model expressiveness.

**Figure 8:**
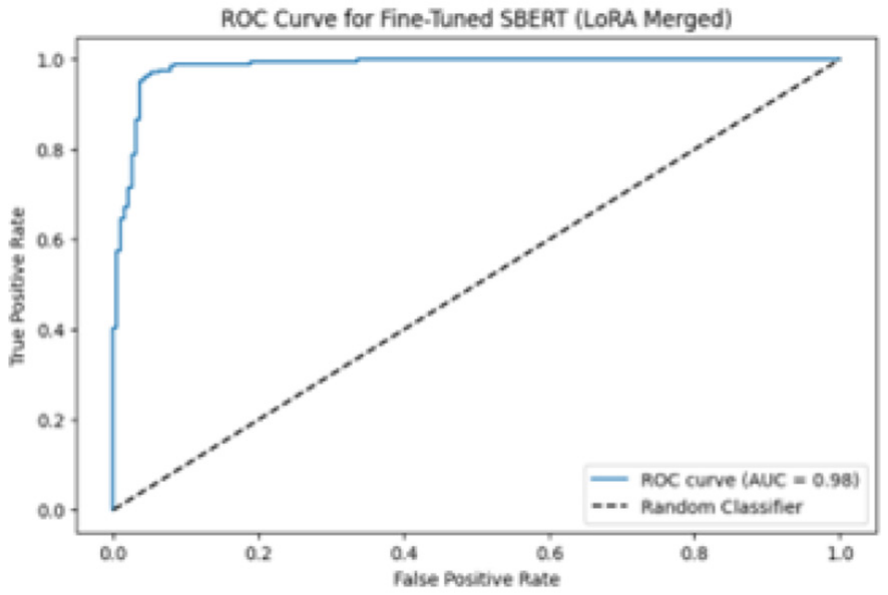
ROC curve for SBERT LoRA.

**Figure 9:**
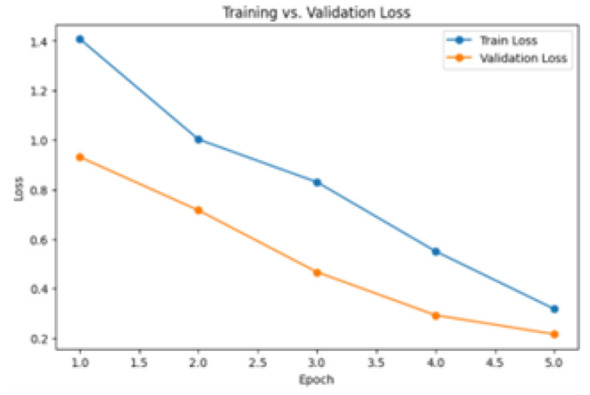
Training vs. validation loss for SBERT LoRA.

LoRA fine-tuned KSI yielded an AUROC value of 0.98 (Figure 8). The training and validation loss curves (Figure 9) show a constant decrease across all the 5 epochs, indicating steady learning and minimal overfitting. The initial training losses exceed those of traditional fine-tuning, yet the system maintains strong performance in the final round of training. In the validation phase (Table 9), the fine-tuned KSI model performed exceptionally well. The LoRA-fine-tuned model delivered comparable results while significantly reducing computational overhead and inference time. The decrease in validation loss indicates the model's generalization capacity. The LoRA-fine-tuned model achieved an accuracy of 81.24%, whereas the fine-tuned model achieved 84.29%. However, recall and F1 scores were notably lower for the LoRA-based model. While the LoRA model is precise in its predictions, we observe that it tends to be conservative in identifying all relevant key sentences. Based on the analysis of the results, we conclude that the standard fine-tuned SBERT offers more balanced performance across all metrics.

**Table 9:** Comparison of LoRA vs. SFT KSI.

Evaluation metrics	LoRA based KSI	SFT based KSI
Accuracy	81.24%	84.29%
Precision	0.9286	0.9501
Recall	0.7343	0.7853
F1 score	0.8206	0.8601
Mean cosine similarity	0.7948	0.8386

.

The performance of the proposed KSI model is further evaluated by comparing it against several well-known baseline methods.

#### 5.4.3 Comparison of KSI with baseline methods

To evaluate the performance of the proposed KSI model, we compare it with several baseline methods commonly used in extractive text summarization. These methods are selected as they represent different approaches to sentence selection and do not rely on domain-specific fine-tuning. The baseline methods considered in this study are:

- **TextRank** (Mihalcea and Tarau, 2004): A graph-based method in which sentences are represented as nodes, and their importance is computed using a PageRank-style algorithm based on sentence similarity.
- **LexRank** (Erkan and Radev, 2004): Similar to TextRank, this method uses a similarity graph and ranks sentences based on eigenvector centrality.
- **Maximal marginal relevance (MMR)** (Carbonell and Goldstein, 1998): A method that selects sentences by balancing relevance with diversity, reducing redundancy in the selected content.
- **Lead-K** (Nenkova and McKeown, 2012): A method that selects the first K sentences from a document, based on the assumption that important information appears early.

These baselines cover graph-based, diversity-aware, and heuristic approaches, providing a useful reference point for evaluating the effectiveness of the proposed KSI model.

Our KSI model performs well when compared with the baseline models such as TextRank, LexRank, Maximal Marginal Relevance (MMR), and Lead-K. We compare the baseline models under an *Oracle-K* setting, where the number of selected sentences *K* is determined by the ground truth. This setting eliminates the bias in the comparison. Table 10 presents the scores for all models.

**Table 10:** Comparison of KSI with baseline models.

Metric	TextRank	LexRank	MMR	Lead-K	SFT-based KSI (Proposed)
Accuracy	68.35%	70.68%	69.50%	60.00%	**84.29%**
Precision	0.7350	0.7555	0.7480	0.6762	**0.9501**
Recall	0.7095	0.7310	0.7200	0.6636	**0.7853**
F1 score	0.7220	0.7430	0.7337	0.6698	**0.8601**

Bold values indicate the highest performance model across the evaluated metrics.

Our KSI obtained a high score across all evaluation metrics. The KSI model performs well with an accuracy of 84.29% and an F1 score of 0.8601. LexRank achieves an accuracy of 70.68% and an F1 score of 0.7430. TextRank comes third with an accuracy of 68.35% and an F1 score of 0.7220. MMR has an accuracy of 60.00% and an F1 score of 0.7337. Lead-K has an accuracy of 60.00% and an F1 score of 0.6698.

Fusion embedding and the dynamic thresholding strategy of KSI justify the good performance of our model over the other models. TextRank and LexRank rely on inter-sentence similarity. Lead-K and MMR depend on positional bias. All baseline methods remain constrained by their reliance on surface-level lexical similarity. Conversely, KSI leverages the fusion of contextual and semantic embeddings, along with adaptive thresholding.

## 6 Conclusion

Thus, given the student responses, our KSI model extracts contextually relevant key sentences. Since this is a proof-of-concept study, we have restricted it to the Operating Systems domain. Our model combines fine-tuned BERT for keyword extraction with fine-tuned SBERT for sentence-level classification. With embedding fusion and dynamic thresholding, our model is trained on a curated dataset. Our experimental results justify that our model performs well when compared with standard baseline models. The results demonstrate that the model is a feasible assistive mechanism for supporting human evaluation.

The main limitation of our study is the lack of reproducibility across different domains. At present, the model is trained on a domain-specific curated dataset. As future work, the dataset can be extended to cover more diverse domains with a structured approach to dataset construction and evaluation. Extending the model in this manner will help in generalizing it to real-world examination scenarios.

## Data Availability

The datasets presented in this study can be found in online repositories. The names of the repository/repositories and accession number(s) can be found below: https://github.com/VeerViswajit/Identification-of-key-sentences.
